# Atypical Papillary Dysplasia of the Bladder Neck

**DOI:** 10.7759/cureus.52726

**Published:** 2024-01-22

**Authors:** Nikit Venishetty, Meesha Trivedi, Jeffrey Annabi, Angelica Padilla, Hani Annabi

**Affiliations:** 1 Department of Urology, Texas Tech University Health Sciences Center El Paso, Paul L. Foster School of Medicine, El Paso, USA; 2 Department of Pathology, Texas Tech University Health Sciences Center El Paso, Paul L. Foster School of Medicine, El Paso, USA

**Keywords:** transurethral resection of bladder tumor (turbt), atypical dysplastic lesions, dysplasia, bladder cancer, papillary dysplasia

## Abstract

As the fourth most frequent disease in men, bladder cancer has a significant financial impact on healthcare. Because atypical dysplasia and papillary forms in bladder cancer are uncommon, there is a dearth of information on them. This study attempts to fill that gap. In the case study that is being presented, a 65-year-old man with a history of prostate cancer was admitted due to unusual urine cytology results that showed bladder papillary atypia. A distinct lesion on the bladder's dome that resembled a raspberry color was discovered by cystoscopy and transurethral resection of the bladder tumor (TURBT), which led to numerous biopsies and resections. Pathology demonstrated a significant urothelial proliferation. The study highlights the variety of morphologies found in atypical dysplastic lesions and the possibility that these lesions could develop into cancer. The significance of identifying atypical dysplastic lesions is emphasized in the study's conclusion, notably in patients with a history of prostate cancer, and highlights the need for further investigation in this domain.

## Introduction

Bladder cancer is the fourth most common cancer among men and is met with high costs of care [1]. The disease represents 3% of all new cancer diagnoses, and over 82,000 cases of bladder cancer were diagnosed in 2023 in the United States, with over 17,000 of those cases leading to death [2]. Bladder cancer can arise from various precursors, including flat urothelial lesions, papillary urothelial lesions, squamous lesions, and glandular lesions [3]. Typical papillary dysplasia, a precursor to low-grade papillary urothelial neoplasms, consists of cytologically benign undulating folds of the urothelium [4]. According to follow-up studies, dysplasia may indicate urothelial instability and disease progression in as many as 19% of patients, indicating the need for these patients to have an active clinical follow-up [5].

Primary dysplasia usually occurs in middle-aged to older patients and predominantly affects males [6]. Macroscopically, it has been described to be an erythematous patch or being cystoscopically unremarkable [6]. However, there has been a paucity of available research on atypical dysplasia or papillary formations due to the rare occurrence of the pathology. The purpose of this case was to describe an atypical dysplasia noted on the dome of the bladder in a patient with a history of prostate adenocarcinoma. The distinct color and non-malignancy of the lesion warrant future studies dedicated to analyzing intricate papillary lesions.

## Case presentation

A 65-year-old male with a past medical history of prostate adenocarcinoma, hypertension, and diabetes and status post radical prostatectomy was admitted for atypical urine cytology with papillary formation occupying the left lateral area of the bladder and bladder neck highly. The patient endorsed trace hematuria. However, the patient denied any suprapubic pain, urinary/voiding changes, dysuria, flank pain, fever, chills, nausea, or vomiting.

On physical exam, there was no suprapubic tenderness, no hernias or adenopathy noted, and no signs of epididymitis, orchitis, hydrocele, or varicoceles. The prostate was noted as flat with no masses. Preoperatively, the Foley catheter revealed light-pink urine. On social history, the patient denied ever smoking and the consumption of alcohol. He was afebrile and vitally stable (Table 1). CBC revealed elevated white blood counts and elevated hematocrits. Red blood cells, hemoglobin, and platelets were all normal. Electrolytes were also normal (Table 1).

**Table 1:** Patient characteristics ALT: alanine transaminase, AST: aspartate transaminase, ALP: alkaline phosphatase

Serum	Patient	Reference
ALT	27 U/L	10-40 U/L
AST	28 U/L	12-38 U/L
ALP	81 U/L	25-100 U/L
Total bilirubin	0.6 mg/dL	0.1-1 mg/dL
Total protein	6.6 g/dL	6-7.8 g/dL
Albumin	3.7 g/dL	3.5-5.5 g/dL
Glucose	234 mg/dL	70-110 mg/dL
Magnesium	1.3 mEq/L	1.5-2 mEq/L
Sodium	136 mEq/L	136-146 mEq/L
Hematologic		
Leukocyte count (WBC)	14,496/mm^3^	4,500-11,000/mm^3^
Hemoglobin	15.5 g/dL	12-16 g/dL (male)
Hematocrit	47.1%	36%-46% (male)

Due to his abnormal urine cytology and abnormal outpatient cystoscopy, the patient was admitted due to a possible neoplasm of the bladder. Due to his history of prostate adenocarcinoma and previous radical prostatectomy, a cystoscopy, transurethral resection of bladder tumor (TURBT), and a biopsy was conducted.

**Figure 1:**
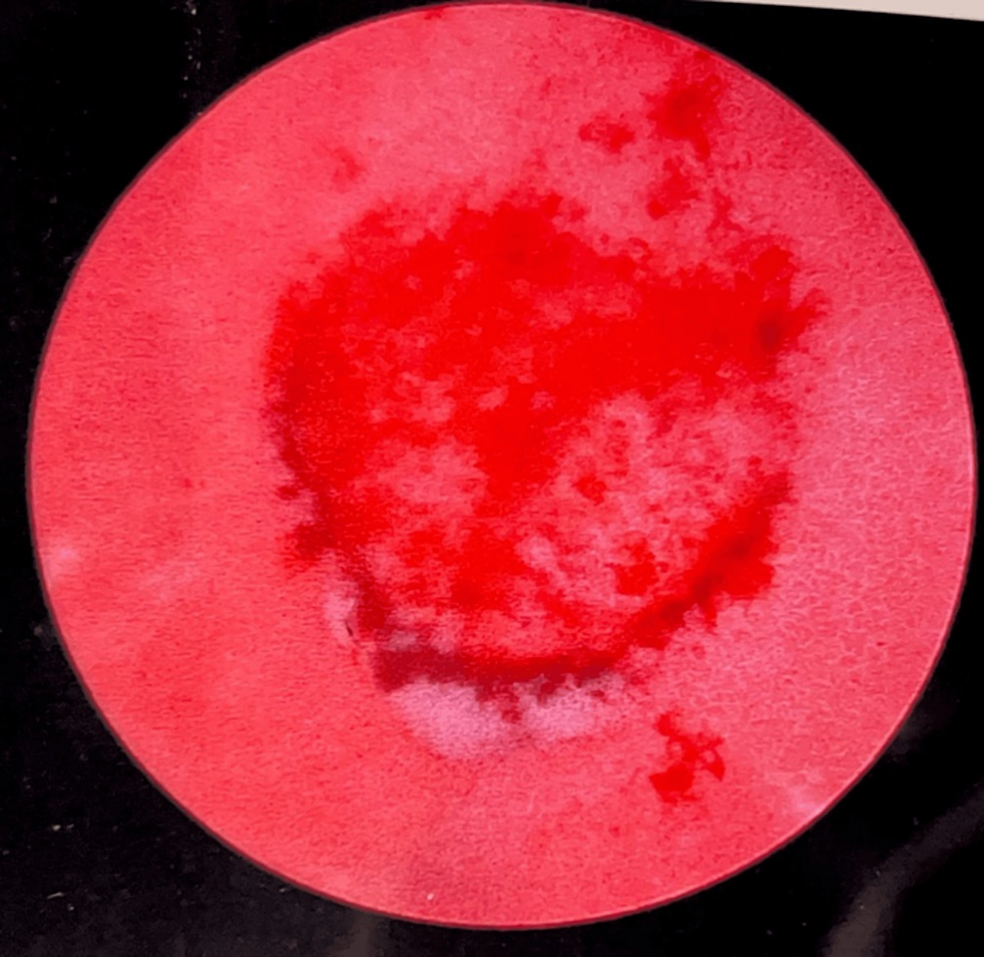
A neobladder revealed a raspberry-like lesion (0.2 cm x 0.6 cm) to the right of the bladder dome, accompanied by a surrounding glandular formation.

Cystoscopy revealed a normal anterior urethra and a missing prostatic urethra. The neobladder was encountered with a lesion resembling a raspberry to the right of the dome of the bladder (Figure 1). The size of the lesion was approximately 0.2 cm x 0.6 cm. There was glandular formation surrounding the lesion. After multiple biopsies were taken, the entire area was resected and fulgurated.

**Figure 2:**
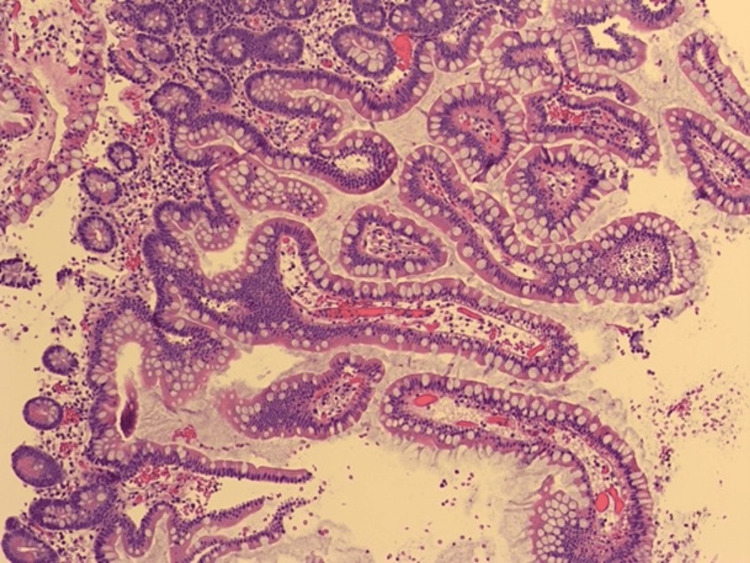
High magnification showing the occasional focus of mild epithelial atypia with a degenerative appearance of the nuclei (40x).

Grossly, the specimen consisted of eight soft pieces of tissue, tan-pink in color, ranging in size from 0.1 to 0.4 cm. Pathology demonstrated a urothelial proliferation with increased cellularity, nuclear enlargement, hyperchromasia, irregular nuclear contours, and interconnected nests (Figure 2). The lesion was well circumcized, and there was neither any exophytic papillary component nor keratinizing squamous differentiation (Figure 2).

Post-operatively, the patient had post-resection hematuria and blood clots, and the bladder was continuously irrigated. However, these episodes resolved and the patient was discharged home with a Foley catheter and a follow-up with Urology.

## Discussion

Bladder cancer has taken on more significance in the field of clinical urology, with its increased incidence likely attributable to the increase in urological complications worldwide [2]. Despite its prevalence, advancements in early detection, robotic surgical techniques, and the evolution of immunotherapy have significantly elevated survival rates [7]. Thus, it has increasingly become more important to identify precursor lesions, such as atypical papillary formations [4]. We present a case of a patient with an abnormal pathology and hope this informs providers of the various morphologies of atypical dysplastic lesions that can occur.

Intravesical chemotherapy, involving the instillation of chemotherapeutic agents directly into the bladder, has been used since the 1960s to treat recurrent superficial bladder cancer [8]. Interleukin-2 as an instillation treatment for superficial bladder cancers has a limited history. Vascular endothelial growth factor (VEGF) inhibitors, which target angiogenesis, may be used to treat or prevent the recurrence of dysplastic lesions [9]. When treating exophytic tumors and visible carcinoma in situ lesions, local surgery is recommended [10]. TURBT is the primary treatment for visible lesions, typically performed under regional or general anesthesia [11]. Due to the atypical formation on the bladder neck in this patient, TURBT was the best practice to decrease any progression to malignancy.

Atypical papillary hyperplasia, which progresses to high-grade papillary cancer, can develop from dysplasia [4]. Thus, it is important to assess any dysplastic lesions present in patients promptly. While lesions can manifest in diverse forms, the exceptional rarity and significance of the raspberry-like lesion in this patient lie in its distinctive and noteworthy gross morphological features.

## Conclusions

Our case report concludes by emphasizing the importance of recognizing atypical dysplastic lesions in patients, especially if they have a history of prostate cancer. Being the fourth most frequent cancer in males, bladder cancer emphasizes how crucial it is to identify precursor lesions early and treat them to reduce the need for ongoing care and related expenses. Atypical dysplasia and papillary forms are uncommon, which complicates our understanding of them and calls for more in-depth investigation and focused studies.
